# Mesenchymal Stem Cells-Derived Exosomes Ameliorate Ischemia/Reperfusion Induced Acute Kidney Injury in a Porcine Model

**DOI:** 10.3389/fcell.2022.899869

**Published:** 2022-05-24

**Authors:** Jianni Huang, Hao Cao, Binbin Cui, Xiaoyan Ma, Ling Gao, Chao Yu, Fengchen Shen, Xinyu Yang, Na Liu, Andong Qiu, Guangyan Cai, Shougang Zhuang

**Affiliations:** ^1^ Department of Nephrology, Shanghai East Hospital, Tongji University School of Medicine, Shanghai, China; ^2^ Department of Cardiac Surgery, Shanghai East Hospital, Tongji University School of Medicine, Shanghai, China; ^3^ Translational Medical Center for Stem Cell Therapy and Institute for Regenerative Medicine, Shanghai East Hospital, Tongji University School of Medicine, Shanghai, China; ^4^ School of Life Science and Technology, Advanced Institute of Translational Medicine, Tongji University, Shanghai, China; ^5^ Department of Nephrology, Chinese PLA General Hospital, Beijing, China

**Keywords:** exosomes, ischemia/reperfusion, acute kidney injury, human umbilical cord derived mesenchymal stem cells, apoptosis, necroptosis, transcriptional factors, macrophages

## Abstract

Exosomes are membrane-enclosed vesicles secreted by cells, containing a variety of biologically active ingredients including proteins, nucleic acids and lipids. In this study, we investigated the therapeutic effects of the exosomes and underlying mechanisms in a miniature pig model of ischemia/reperfusion-induced acute kidney injury (I/R-AKI). The exosomes were extracted from cultured human umbilical cord derived mesenchymal stem cells (hUC-MSCs) and infused into a miniature pig model of I/R AKI. Our results showed that 120 min of unilateral ischemia followed by reperfusion and contralateral nephrectomy resulted in renal dysfunction, severe kidney damage, apoptosis and necroptosis. Intravenous infusion of one dose of exosomes collected from about 4 × 10^8^ hUC-MSCs significantly improved renal function and reduced apoptosis and necroptosis. Administration of hUC-MSC exosomes also reduced the expression of some pro-inflammatory cytokines/chemokines, decreased infiltration of macrophages to the injured kidneys and suppressed the phosphorylation of nuclear factor-κB and signal transducer and activator of transcription 3, two transcriptional factors related to inflammatory regulation. Moreover, hUC-MSC exosomes could promote proliferation of renal tubular cells, angiogenesis and upregulation of Klotho and Bone Morphogenetic Protein 7, two renoprotective molecules and vascular endothelial growth factor A and its receptor. Collectively, our results suggest that injection of hUC-MSC exosomes could ameliorate I/R-AKI and accelerate renal tubular cell repair and regeneration, and that hUC-MSC exosomes may be used as a potential biological therapy for Acute kidney injury patients.

## Introduction

Acute kidney injury (AKI) is characterized by an abrupt increase in serum creatinine concentration and/or a sudden decrease of urine output, both occurring within 48 h or less (Chawla et al.,2017). In a population-based epidemiology study, the rate of AKI in the intensive care unit was reported at 16.92%, and the incidence of AKI in the general ward was 6.72% (Kashani et al., 2017). The effect of AKI on individual outcomes and societal burden is remarkable (Lewington et al., 2013), Thus, improved prevention and treatment of AKI would overcome many obstacles to improving outcomes among hospitalized patients. Despite many clinical trials of possible interventions, there are still no reliable and specific means to prevent or reverse AKI; treatment still relies on hemodynamic optimization, avoidance of nephrotoxicity and renal replacement therapy if all else fails and there is no timely recovery (Pickkers et al., 2017). Thus, it is critical to further explore new approaches to treat this disease.

AKI can be induced by multiple pathological conditions, such as ischemia-reperfusion (I/R), sepsis, trauma, and nephrotoxins (Lameire et al., 2013; Ni et al., 2019). Renal I/R is one of the most common causes of AKI. Following ischemic injury, renal tubular cells, in particular proximal tubular cells develop various types of cell death. Apoptotic and necrotic cell death are two common types of renal tubular epithelial cell death (Linkermann et al., 2013; Ni et al., 2019). Apoptosis is predominantly mediated by activation of caspases such as caspase-3 (Ni et al., 2019) and the disruption of mitochondrial fission-fusion dynamics and loss of epithelial cell integrity (Yan et al., 2020). Necroptosis is characterized by phosphorylation of receptor-interacting protein kinase 3 (RIPK3) and RIPK3-mediated phosphorylation of the pseudo-kinase mixed lineage kinase domain-like protein (MLKL). High expression levels of RIPK3 promote RIPK3 oligomers aggregating and forming a higher-order structure (i.e., necrosome) that leads to MLKL phosphorylation (Martin-Sanchez et al., 2017). Furthermore, the inflammatory response amplifies kidney injury and tubular cell death (Martin-Sanchez et al., 2017). There are multiple interactions between inflammation and apoptosis/necroptosis. For example, activation of necroptosis can promote the expression of some pro-inflammatory factors such as monocyte chemoattractant protein-1 (MCP-1) and tumor necrosis factor-α (TNF-α) through necroinflammation (Mulay et al., 2016). Meanwhile, the kidney has a remarkable potential to repair AKI through renal tubular cell regeneration and preservation of the peritubular vascular network. These processes are involved in preservation of some renoprotective factors like Klotho (Liu et al., 2019) or Bone Morphogenetic Protein 7 (BMP-7) (Vigolo et al., 2019) and the production of some pro-angiogenic cytokines such as VEGFA (Chang et al., 2021; Medica et al., 2021). Recently, it has been reported that extracellular vesicles from diverse cells including mesenchymal stromal cells (MSCs) can offer renoprotection and promote renal regeneration following AKI and some chronic insults.

In the past decade, the therapeutic potential of MSCs for AKI has been extensively recognized (Humphreys and Bonventre, 2008). MSCs exert immunomodulation, anti-apoptotic, antioxidative, and pro-angiogenic effects mainly through endocrine and/or paracrine means (Selim et al., 2019; Fazekas and Griffin, 2020). Extracellular vesicles (EV) are encapsulated bio-membranes that are shed by cells into extracellular milieu and contain a varied biomolecule cargo, including saccharides, proteins, lipids, and nucleic acids (Soekmadji et al., 2020). Exosomes are the main mediators of intercellular communication in EVs, and they distinguish themselves by biophysical and biochemical characteristics and size (50–150 nm in diameter) from other EV such as microvesicles (100–1,000 nm) or apoptosis body (50–500 nm) (Thery et al., 2009).

Recently, Liu et al. have reported that injection of MSCs derived EV to a murine model with I/R-induced AKI revealed a renoprotective effect (Tang et al., 2020; Cao et al., 2021). However, the physiological differences between human and small animals often limit the translation of laboratory data to clinical applications (Huang et al., 2021). Body size and physiological patterns of kidney injury in a miniature pig more closely mimic those described in humans, which makes this larger animal more attractive for verifying or identifying novel therapeutic agents in the field of biological medicine. Therefore, the purpose of this study was to verify the efficacy of MSCs-derived exosomes in miniature pig model of AKI induced by I/R and further explore the mechanism by which exosomes offer renoprotection in this model.

## Materials and Methods

### Materials

Human umbilical cord MSCs (hUC-MSCs) were obtained from the GMP laboratory of Shanghai East Hospital, Tongji University School of Medicine, Shanghai, China. Bama miniature pigs (*Sus scrofa*) were purchased from Taihe Biotechnology Co., Ltd. (Taizhou, Jiangsu, China). Antibodies to Cleaved Caspase 3, Phospho-MLKL, CD9, CD81, Phospho- NF-κB p65, Phospho- NF-κB p65, STAT 3, Phospho- STAT 3, BMP 7 and α-Tubulin were purchased from Cell Signaling Technology (Danvers, MA, United States). An antibody to phospho-RIPK3 was purchased from Abcam (Cambridge, United Kingdom). Antibodies to NF-κB p65 and Klotho were purchased from Santa Cruz Biotechnology (Santa Cruz, CA, United States). Antibodies to MLKL, RIPK3, F4/80, VEGFR2 were obtained from Proteintech (Wuhan, China). Antibodies to VEGFA and CD31 were obtained from Invitrogen (Carlsbad, CA, United States). Antibodies to GAPDH and PCNA were obtained from Arigo (Taipei, Taiwan, China). An antibody to NGAL was purchased from Genetex (San Antonio, TX, United States).

### Isolation and Characterization of Human MSC-Derived Exosomes

MSCs were cultured with α-MEM (Gibco) supplemented with 5% serum substitute UltraGRO™ Advanced (Helios). MSCs (Passage 6) were cultured with FBS-free media for 72 h, and the supernatants were subsequently collected for exosomes extraction. Exosomes were isolated by ultracentrifuge and filtration as previously described (Cao et al., 2021). In brief, the supernatants were subject to gradient centrifugation at 300×*g* for 20 min and 2000×*g* for 30 min, and then filtered with a 0.22-μm filter (Millipore) and ultracentrifuged at 120,000×*g* for 150 min (Beckman Coulter Optima XPN-100) at 4°C. For each preparation, 8 tubes (26 ml/tube) of cell medium supernatant from 4 × 10^7^ hUC-MSCs was ultracentrifuged. The exosome pellets were resuspended in sterile phosphate buffer saline (PBS) and filtered using a 0.22-μm filter once again. The above solution at approximately 15 ml was then condensed using Amicon^®^ Ultra 100 K centrifugal filter (Millipore), resulting in about 500 μl of exosomes in the PBS. In selected experiments, exosomes were directly labelled with 1 μM red fluorescent dye DiI (Shanghai, Beyotime) during the ultracentrifugation procedure and then washed twice by PBS to remove the excess dye (Grange et al., 2014). Exosomes were stored at −80°C until use.

Before injection to pigs, total protein concentration in exosomes lysates was measured using a BCA kit (Shanghai, Beyotime), and exosomal surface markers (CD9 and CD81) were analyzed by immunoblot analysis. We evaluated exosome concentration, shape, and size by transmission electron microscopy and Nanoparticle Tracking Analysis (NTA; ZetaVIEW S/N 252) as previously described (Medica et al., 2021). The major organs of pigs infused with DiI-labeled exosomes were frozen sectioned, and the distribution of exosomes was evaluated under fluorescence microscope.

### Animals Models of AKI and Treatment

Thirty-two female Bama miniature pigs weighed 17.65 ± 2.22 kg were quarantined and allowed to acclimatize for 2 weeks before experimentation. Pigs were fed standard diet (Synergetic biology, Jiangsu, China) twice a day, and had free access to municipal tap water. In the modeling stage, 16 pigs were randomly divided into 4 groups, and subjected to ischemia by claiming for 0, 60, 90, and 120 min, respectively. After preliminary experiments, 120 min-ischemia was chosen as the standard time to create the model for administration of exosomes. Miniature pigs were randomly assigned to 4 groups: 1) sham + vehicle; 2) sham + exosomes; 3) I/R + vehicle; and 4) I/R + exosomes groups. There were four pigs in each group. Fasting for 24 h before surgery.

Anesthesia was induced by intramuscular injection of Zoletil 50 (Virbac, France) before surgery. For each pig, tracheal intubation was administered, intravenous indwelling needle was inserted in the ear and 1 mg/kg of heparin sodium was infused intravenously (systemic heparinization). The pig was placed in a supine position, connected to a ventilator and an intensive ECG monitor, 2% isoflurane was used to maintain anesthesia, and succinylcholine chloride (Shanghai XudongHaipu Pharmaceutical Co., Ltd.) was used as a muscle relaxant. A 10 cm incision on the midline of the abdomen was made to expose both kidneys. The right renal pedicle was first exposed and ligated followed by a right nephrectomy. Then, the left renal artery was separated and ligated with a non-destructive vascular ligation band and a Lumier trocar in I/R groups. In the control group, the renal artery was isolated without ligation. MSCs-derived exosomes (1 × 10^9^ particles/kg) or equal volume of PBS were injected intravenously during the ischemia. The dose of exosomes was determined on the basis of previous studies on mouse model by the equivalent dose conversion method between different species in pharmacological experiments. After surgery, blood samples were taken from porcine anterior vena cava to assess renal function as indicated in figure legends. 72 h after reperfusion, the left kidney was removed under anesthesia. The kidney tissue at the junction of the cortex and medulla was snap-frozen and fixed with 4% paraformaldehyde for further research. Potassium chloride was injected intravenously to euthanize the pig.

### Renal Function Analysis

Serum creatinine (SCr) and blood urea nitrogen (BUN) levels were determined by a Creatinine Assay Kit and a BUN kit (Nanjing Jiancheng Bioengineering Institute, Nanjing, China), respectively, in accordance with the manufacturer’s instructions.

### Immunoblot Analysis

To prepare protein samples for western blotting, kidney tissue samples were homogenized in the presence of RIPA (Shanghai, Beyotime) and a protease inhibitor cocktail (Shanghai, Beyotime). In brief, a total of 20 µg protein were separated by SDS-PAGE gel electrophoresis and transferred to PVDF membrane in a tank (Tenon Electrophoresis System, China). The membrane was blocked with 5% nonfat milk for 1 h at room temperature while shaking, and then incubated with specific primary antibodies at 4°C overnight. After being washed three times in Tris Buffered Saline Tween (TBST), the membrane was incubated with a secondary antibody for 2 h at room temperature while shaking. Followed by TBST rinsing, bound antibodies were visualized by Chemidoc MP imaging system (Biorad).

### Pathological Assessment

The kidney tissues were fixed in 4% paraformaldehyde for paraffin embedding. Hematoxylin and eosin staining were applied in kidney sections (thickness 3 µm) to examine the pathological changes. Deparaffinized sections were stained with a kit according to the protocol provided by the manufacturer and observed by light microscopy. Tubule injury was scored on a scale from 0 to 3, where 0 = normal, 1 = injury area <30%, 2 = 30%–60% of tubules injury, 3 = injury area >60%.

### Immunohistochemistry and Immunofluorescence Staining

Immunohistochemistry and immunofluorescence staining were performed in kidney sections (thickness 3 µm) with various antibodies as seen in Supplementary Table S1 after pre-treated with EnVision^TM^ FLEX Target Retrival Solution (Dako). After the completion of secondary antibody incubation, sections were counterstained with DAPI (Beyotime). and mounted with anti-fluorescence quenching mounting media (Beyotime), or treated with DAB staining solution (Maixin, China). Photographs were blindly taken at random fields under a microscope ×200 magnification using Leica optical Microscope or Leica Automatic Fluorescence Microscope (Leica DFC7000).

### TUNEL Assay

Kidneys were fixed in 4% paraformaldehyde, embedded in paraffin and sectioned. A terminal deoxynucleotidyl transferase mediated dUTP-biotin nick end labeling (TUNEL) assay was performed using One Step TUNEL Apoptosis Assay Kit (Beyotime) following the manufacturer’s protocol. Mounted samples were analyzed by Automatic Fluorescence Microscope (Leica DFC7000).

### RNA Extraction, cDNA Synthesis and Real-Time qPCR

The porcine kidneys were snap frozen in liquid nitrogen after harvesting and kept at −80°C. The frozen kidney was then homogenized and total RNA was isolated, using RNAeasy™ Animal RNA Isolation Kit with Spin Column (Beyotime). PrimeScript™ RT reagent Kit (TAKARA) was used to convert mRNA to cDNA. The concentration of cDNA was measured with NanoDrop One Microvolume UV Vis Spectrophotometer (Thermo Fisher). The expression levels of genes were analyzed by Applied Biosystems 7500 Fast Real-Time PCR System (Applied Biosystems), using TB Green^®^ Premix Ex Taq™ (TAKARA). The relative expression of a gene of interest was normalized to housekeeping gene GAPDH. Sequences of primers used in this study can be seen in Supplementary Table S2.

### Statistical Analyses

Data were presented as mean ± SEM. Statistical analyses were performed using IBM SPSS Statistics 23. Normally distributed data was analyzed using Student’s two-tailed t-tests, or two-way analysis of variance (ANOVA). Non-normally distributed data was analyzed using Kruskall–Wallis nonparametric testing. *p* < 0.05 was considered as statistically significant difference. Graphs were depicted using Prism 8 (GraphPad).

## Results

### Successful Establishment of a Porcine Model of I/R Induced AKI

**FIGURE 1:**
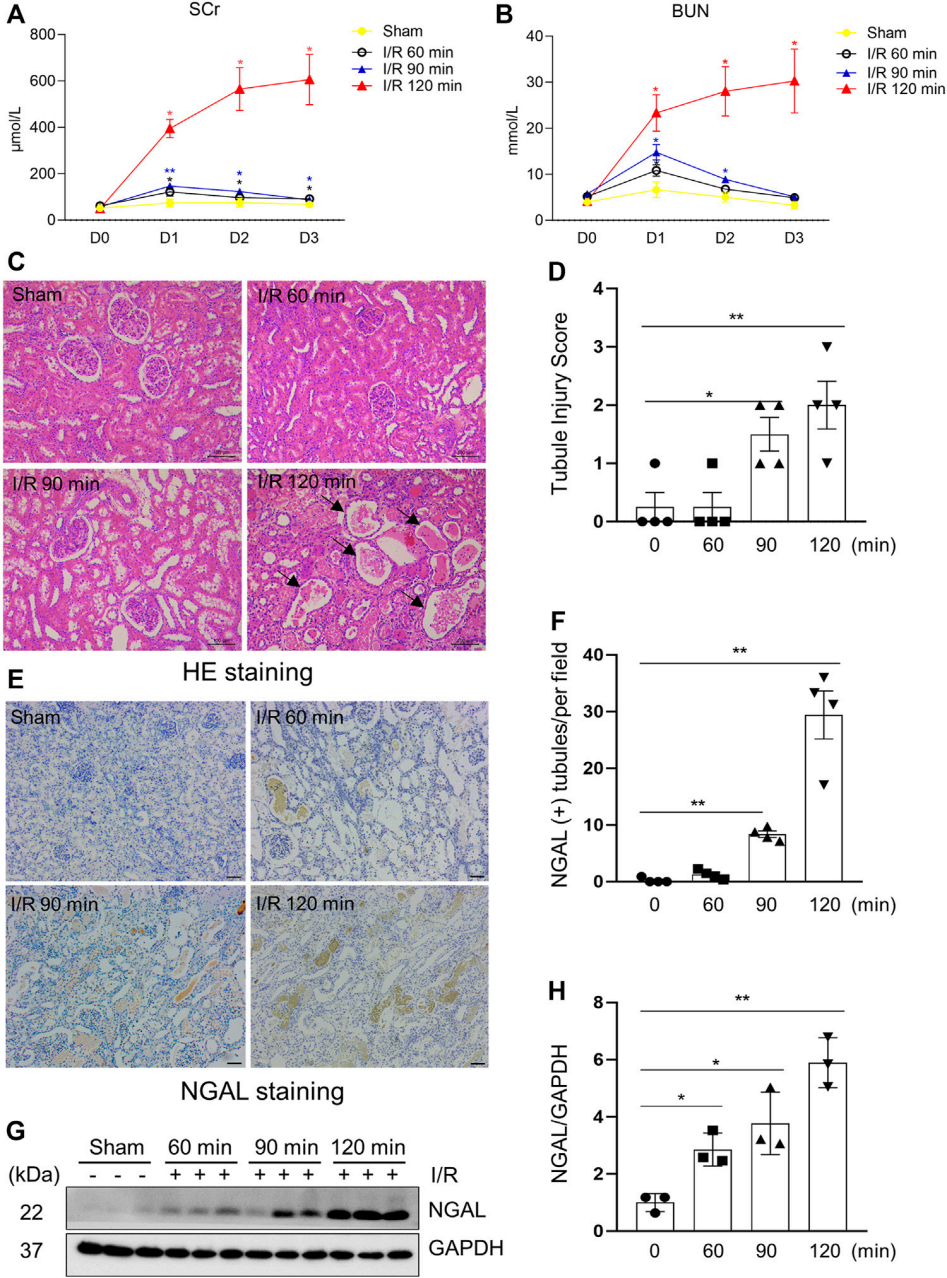
Kidney injury indicators elevated after surgical establishment of I/R Induced AKI in a porcine Model. **(A,B)** Renal function of pigs after different ischemia duration and reperfusion (each group *n* = 4). Blood was sampled before operation and 24, 48, 72 h after reperfusion. Values at different time points vs. baseline value (D0) from the same group for statistical analysis. SCr, serum creatinine; BUN, blood urea nitrogen. **(C)** Photomicrographs illustrating hematoxylin and eosin staining of kidney tissue from each group. Arrows point to injured tubules with intraluminal casts. **(D)** Tubule Injury was scored and graphed to analyze the severity of renal tubular injury. **(E)** Photomicrographs illustrating immunohistochemical staining of NGAL. Scale bar = 100 µm. **(F)** Quantification of the numbers of NGAL positive tubules from immunohistochemical staining. **(G)** The kidney tissue lysates from four groups were subject to immunoblot analysis with specific antibodies against NGAL. **(H)** Expression levels of NGAL were quantified by densitometry analysis and then normalized with GAPDH (3 independent trials). Data are means ± sem.*p < 0.05; **p < 0.01 versus sham controls.

There are no generally accepted standardized procedures for establishing a I/R model in pigs (Baldwin et al., 2004; Bechara et al., 2016; Damasceno-Ferreira et al., 2018). As an initial step towards assessing the efficacy of MSCs derived EV in this model, we first tried to establish a model of I/R-induced AKI by clamping left renal artery using a ligation band for different time (60, 90, 120 min) followed by re-flow and contralateral nephrectomy. Injury to the kidney by ischemia/reperfusion (I/R) led to an increase of serum creatinine (SCr) (Figure 1A) and blood urea nitrogen (BUN) (Figure 1B) in all 3 experimental time groups 24 h post-operation, indicative of developing renal dysfunction. Subsequently, the renal function of miniature pigs in the 60-min and 90-min ischemia groups gradually re-covered to approximate baseline levels within 48 h. However, SCr and BUN in the 120-min ischemia group remained at high levels over the entire 72-h reperfusion time examined in this study (Supplementary Table S3). The reperfusion was initiated by the loosen of ligation band (Supplementary Figure S1).

To investigate the effect of different ischemia time on the severity of kidney damage in miniature pigs, we examined histopathological changes and expression of tubule injury markers in each group. The results from hematoxylin-eosin staining showed that the kidney subjected to a longer ischemic time (120 min) developed more severe renal tubule damage than shorter ischemic time (60, 90 min); Figures 1C–F showed that the kidneys subjected to 120 min of ischemia exhibited obvious granular casts, tubular dilatation, and detachment of tubular cells as well as expressed higher levels of neutrophil gelatinase-associated lipocalin (NGAL), an early biomarker of renal injury (Silberstein et al., 2013). Immunoblot analysis also demonstrated a time-dependent increase of NGAL expression in ischemic kidneys (Figures 1G,H). These results illustrated that 120 min of renal ischemia can stably induce renal tubular damage and AKI in miniature pigs, thereby being applied for subsequent intervention experiments.

### Apoptosis and Necroptosis Occur in Renal Tubular Cells of Miniature Pig of I/R-Induced AKI

**FIGURE 2:**
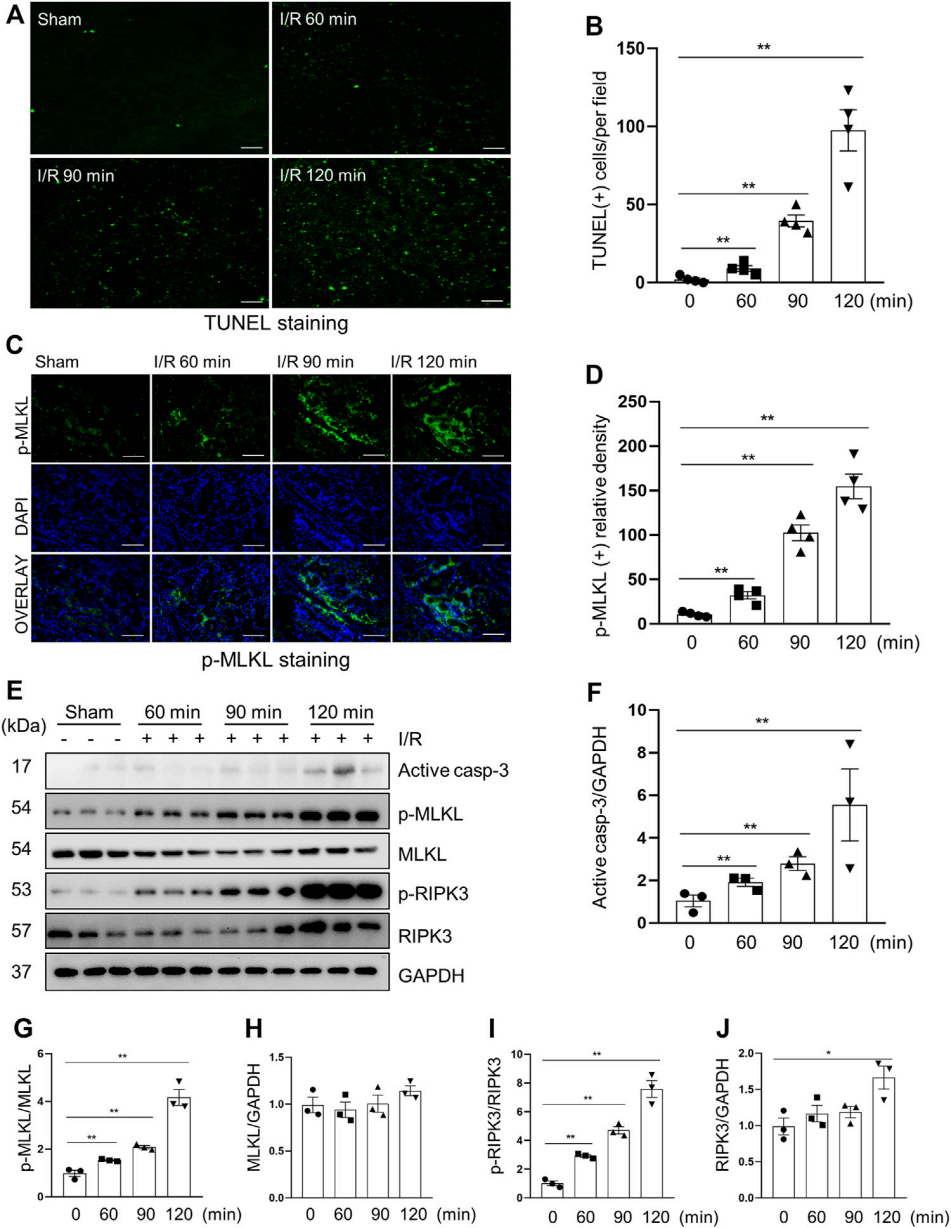
Apoptosis and necroptosis levels in miniature pigs with acute kidney injury induced by ischemia-reperfusion. **(A)** Representative images of TUNEL staining of kidney tissue from each group. Scale bar = 100 µm. **(B)** Quantification and plot of the TUNEL stained images. **(C)** Photomicrographs illustrating immunofluorescence staining of phospho-MLKL. Scale bar = 200 µm. **(D)** Quantification and plot of the immunofluorescence integrated density of phospho-MLKL. **(E)** Porcine kidney tissue lysates from four groups were subject to immunoblot analysis with specific antibodies against cleaved-caspase 3, MLKL, phospho-MLKL, RIPK3 and phospho-RIPK3. Expression levels of cleaved-caspase 3 **(F)**, MLKL **(H)** and RIPK3 **(J)** were quantified by densitometry analysis and then normalized with GAPDH. The phosphorylation levels of MLKL **(G)** and RIPK3 **(I)** were calculated quantitatively by densitometry. Data are means ± sem.*p < 0.05; **p < 0.01 versus sham controls.

Apoptosis and necroptosis are two types of cell death of renal tubular cells and contribute to the pathogenesis of AKI (Anders, 2018; de Jesus-Silva et al., 2020). In the pig model of I/R-induced AKI, we observed apoptotic renal tubular cells, as shown by TUNEL positive staining (Figures 2A,B) and increased expression of cleaved caspase-3, a major apoptotic marker (Figures 2E,F), and necrotic tubular cells, as evidenced by increased phosphorylation levels of MLKL and RIPK3, two markers of necroptosis, by both immunofluorescent staining (Figures 2C,D) and immunoblotting (Figures 2E,G–J). As expected, the degree of apoptosis and necroptosis was increased with the prolonged ischemia. The enhancement of apoptosis was the most significant in the 120-min group, which was consistent with the severity of kidney injury as shown by the expression of NGAL as mentioned above.

### Extraction and Characterization of Exosomes From Human MSCs

**FIGURE 3:**
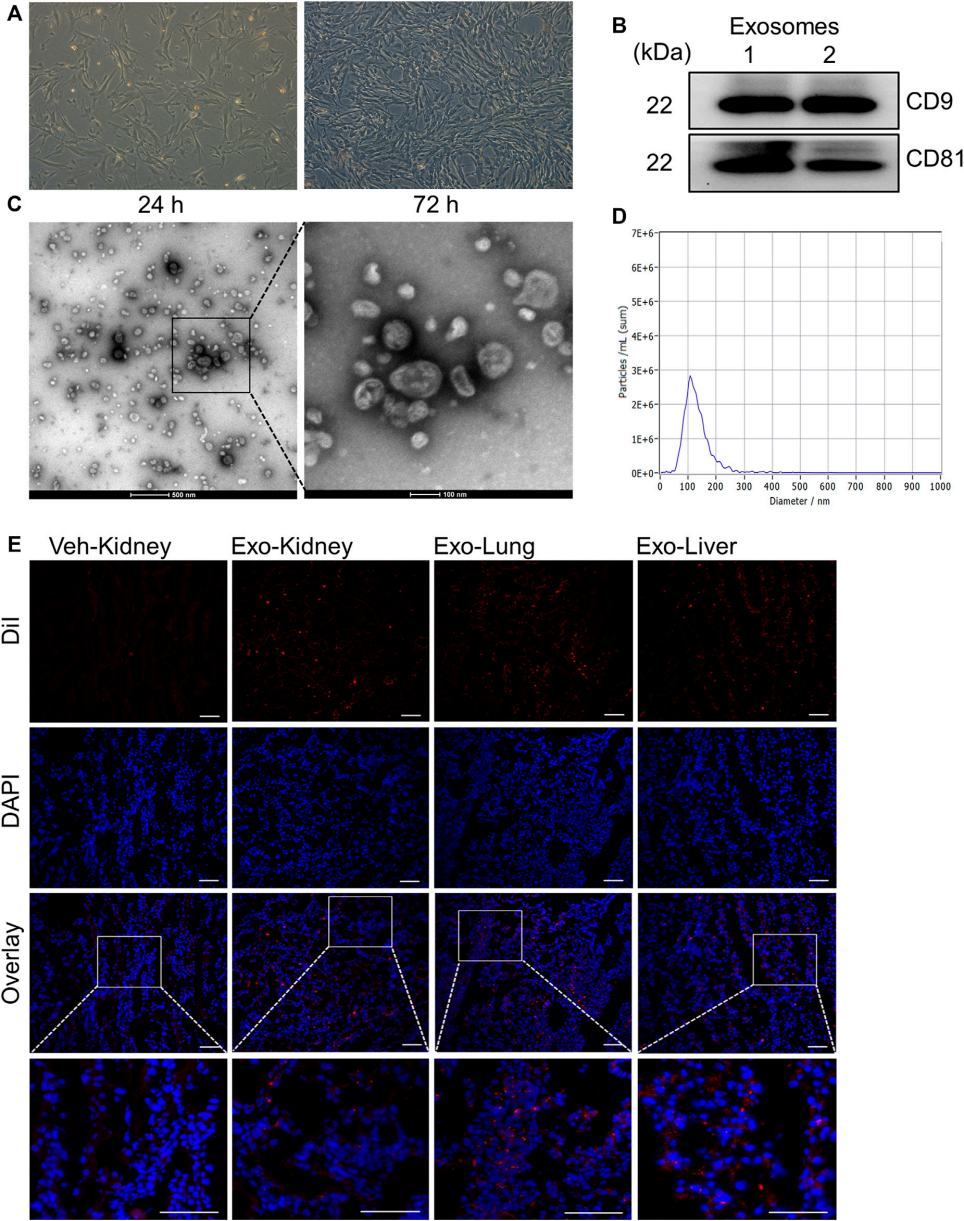
Extraction and identification of exosomes from mesenchymal stem cells derived from human umbilical cord. **(A)** Pictures of the 6th passage of mesenchymal stem cells after 24 and 72 h of culture. Scale bar = 100 µm. **(B)** The expression of the marker molecules CD9 and CD81 in the exosome lysate was detected by immunoblotting. Two samples from different extraction batches was tested. **(C)** Morphology of exosomes observed under transmission electron microscope. **(D)** The particle size distribution of exosomes by Nanoparticle Tracking Analysis. **(E)** Biodistribution of exosomes labeled by DiI in different organs. Scale bar = 100 µm.

All hUC-MSCs used in this study have undergone a full set of tests, which has been proved by reports from China National Institute for Food and Drug Control are available. To assess the effect of exosomes on AKI, we first characterized the exosomes extracted from human umbilical cord derived mesenchymal stem cells (hUC-MSCs). Exosomes were extracted from culture medium supernatants of hUC-MSCs that grown to 80–90% confluence (Figure 3A) by ultracentrifugation. Total protein extracted from exosome lysates at 311.75 ± 54.21 μg/ml was used for immunoblot analysis of exosomal marker proteins CD9 and CD81. Figure 3B shows that both CD9 and CD81 were clearly detected. The extracted exosome samples were further examined by transmission electron microscopy, showing spherical, hemispherical or saucer-shaped vesicles with a diameter of about 100 nm (Figure 3C). Nanoparticle Tracking Analysis (NTA) show that the concentration of exosomes is 4.5 × 10^9^ Particles/ml, and more than 90% of the particles are around 110 nm in diameter (Figure 3D). All these results illustrated that we had extracted pure exosomes from hUC-MSCs.

Next, DiI-labeled MSCs derived exosomes were intravenously injected into I/R and sham-operated pigs injured to examine their distribution *in vivo*. Porcine kidney, heart and lung tissues were taken for frozen sections after the sacrifice. As shown in Figure 3E, the exosomes are distributed in various organs. The fluorescent signals in the kidney of pig with AKI were also higher compared with sham-operated kidneys. Thus, we have successfully extracted the exosomes from hUC-MSCs and observed their distribution in diverse organs, including the kidney.

### MSCs-Derived Exosomes Alleviate Renal I/R Injury in Miniature Pigs

**FIGURE 4:**
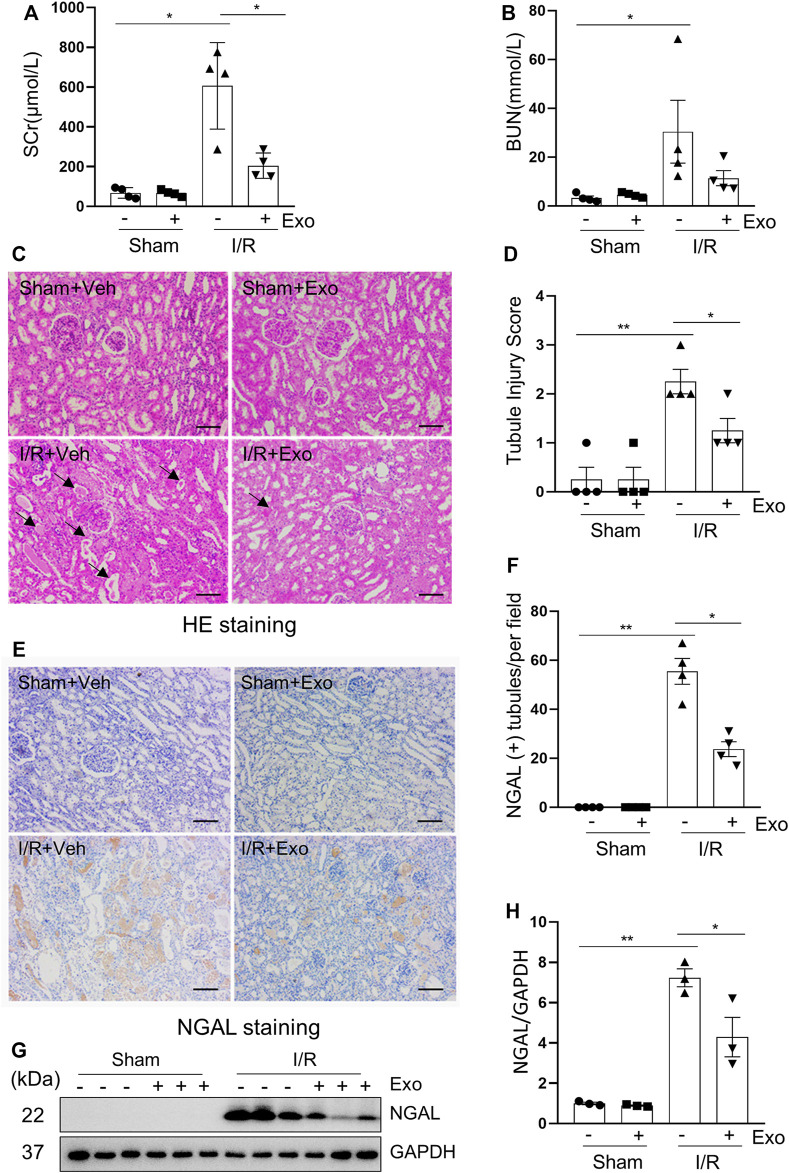
The renal ischemia-reperfusion injury in miniature pigs was significantly reduced after exosomes treatment. **(A,B)** Serum creatinine (SCr) and blood urea nitrogen (BUN) at 72 h after treatment. **(C)** Photomicrographs illustrating hematoxylin and eosin staining of kidney tissue from four groups. Arrows point to injured tubules with intraluminal casts. Scale bar = 100 µm. **(D)** Tubule Injury was scored and graphed to analyze the severity of renal tubular injury. **(E)** Photomicrographs illustrating immunohistochemical staining of NGAL. Scale bar = 100 µm. **(F)** Quantification and plot of the percentage of NGAL positive tubules from immunohistochemical staining. **(G)** Porcine kidney tissue lysates from four groups were subject to immunoblot analysis with specific antibodies against NGAL. **(H)** Expression levels of NGAL were quantified by densitometry analysis and then normalized with GAPDH. Data are means ± sem.*p < 0.05; **p < 0.01 versus sham controls.

To explore the efficacy of hUC-MSCs in I/R-induced AKI, the pig model with 120 min-ischemia followed by 72 h-reperfusion were used. Pigs were injected intravenously with one dose of exosomes (1 × 10^9^ particles/kg) or PBS during the period of renal ischemia. Figures 4A,B shows that I/R-induced SCr and BUN were significantly lower in pigs subjected to exosome injection relative to the group treated with PBS alone (Supplementary Table S4). Pathological changes of the kidney such as interstitial edema, renal tubule dilatation, vacuolar degeneration, necrosis and detachment of epithelial cell and casts formation were significantly mitigated following exosome administration (Figure 4C), with the renal tubular injury score reduced accordingly (Figure 4D). Renal expression levels of NGAL examined by either immunohistochemistry (Figures 4E,F) or immunoblotting (Figures 4G,H) were also decreased in pigs with I/R and exosome injection treatment compared to PBS alone treated pigs. Together, our data indicate that MSCs-derived exosomes have a strong renoprotective effect in the model of I/R-induced AKI in pigs.

### MSCs-Derived Exosomes Inhibited I/R Induced Apoptosis and Necroptosis in the Kidney of Miniature Pigs

**FIGURE 5:**
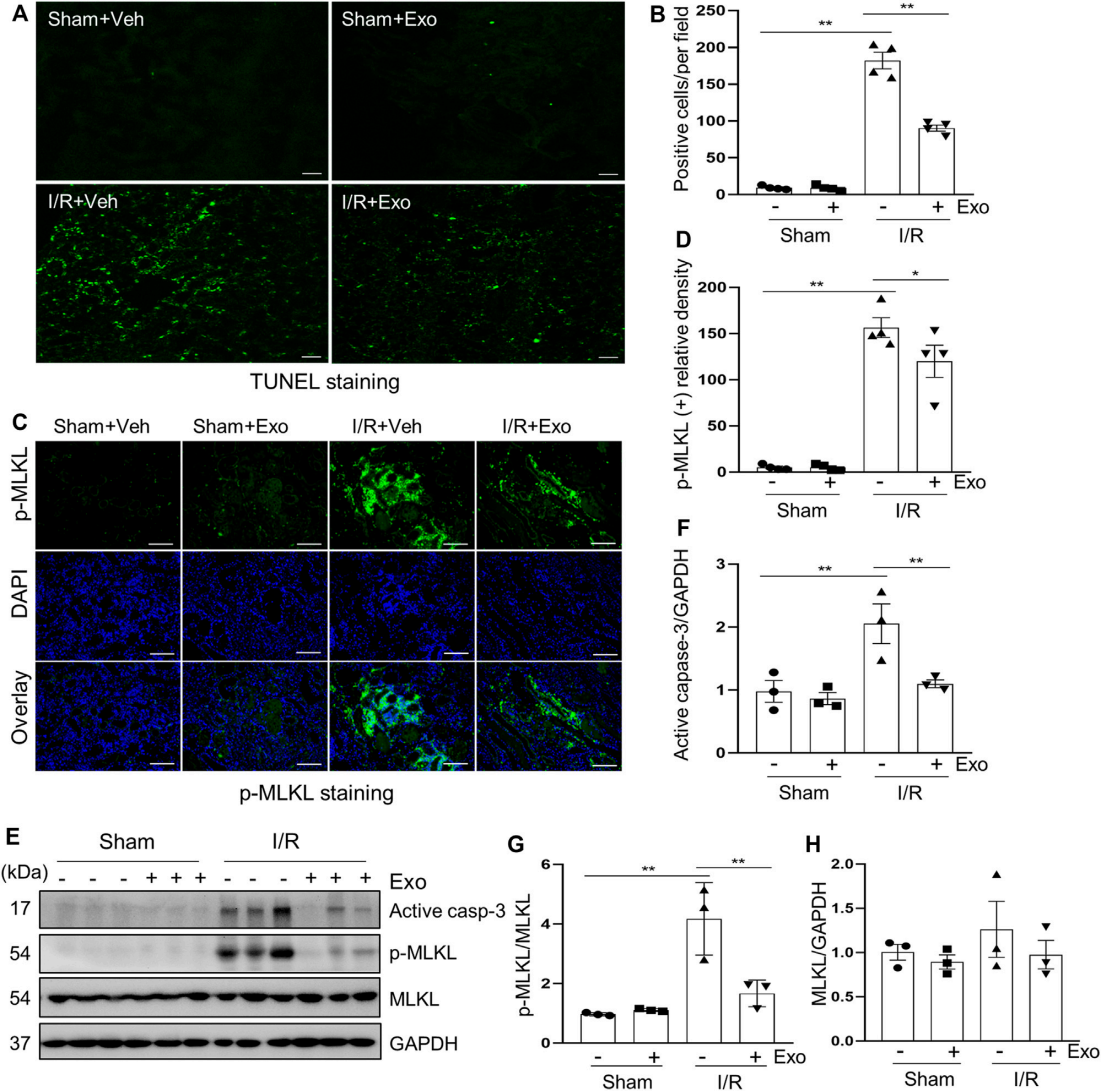
Exosomes down-regulated apoptosis and necroptosis levels in pigs with acute kidney injury induced by ischemia-reperfusion. **(A)** Representative images of TUNEL staining of kidney tissue from four groups. Scale bar = 100 µm. **(B)** Quantification and plot of the TUNEL stained images. **(C)** Photomicrographs illustrating immunofluorescence staining of phospho-MLKL. Scale bar = 200 µm. **(D)** Quantification and plot of the Immunofluorescence integrated density of phospho-MLKL. **(E)** Porcine kidney tissue lysates from four groups were subject to immunoblot analysis with specific antibodies against cleaved-caspase 3, MLKL, phospho-MLKL. Expression levels of cleaved-caspase 3 **(F)** and MLKL **(H)** were quantified by densitometry analysis and then normalized with GAPDH. The phosphorylation level of MLKL **(G)** was calculated quantitatively by densitometry. Data are means ± sem.*p < 0.05; **p < 0.01 versus sham controls.

We further explored the effect of MSCs-derived exosomes on apoptosis and necroptosis in the kidney of pigs following I/R. and demonstrated that exosomes infusion significantly reduced the levels of apoptosis and necroptosis in injured porcine kidneys. This was clearly observed in Figures 5A,B, which shows that the number of the TUNEL-positive cells in the exosome treated group was significantly lower than that in the non-treatment I/R group and in Figures 5E,F, which shows that the expression level of cleaved-caspase 3 was also lower in the exosome treatment group. Similarly, immunofluorescent staining showed that renal ex-pression levels of p-MLKL were reduced in exosome treated pigs (Figures 5C,D). Additionally, I/R-induced expression and phosphorylation of MLKL was suppressed by exosomes as well, as detected by immunoblot analysis (Figures 5E,G,H). The above data suggest that intravenous infusion of MSCs-derived exosomes can attenuate apoptosis and necroptosis of renal tubular cells, which may be an important mechanism for MSCs-derived exosomes to protect against AKI.

### MSCs-Derived Exosomes Promote Renal Regeneration and Expression of Renoprotective Factors in the Kidney of Miniature Pigs Following I/R Injury

**FIGURE 6:**
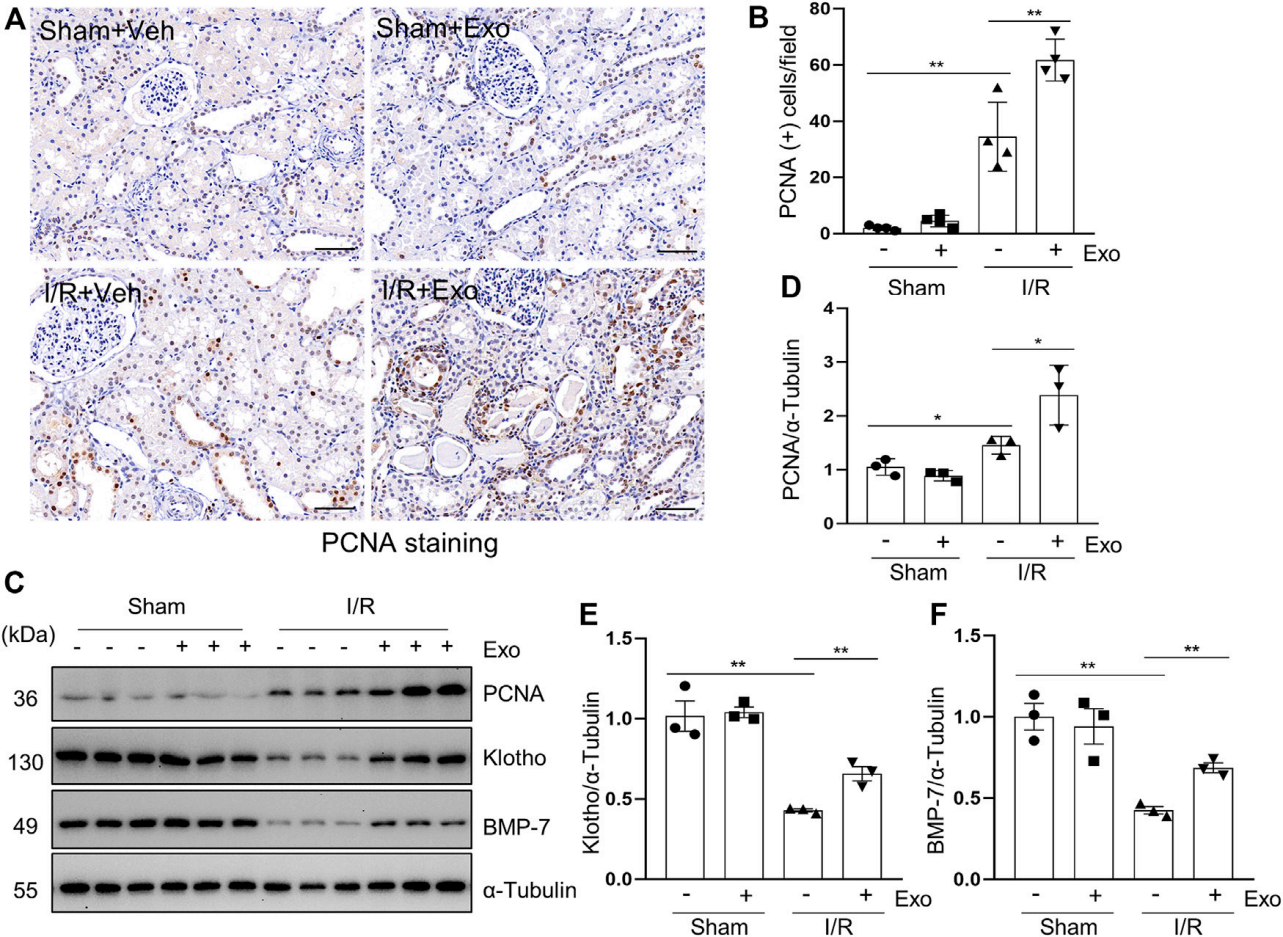
MSCs-derived exosomes promote renal regeneration and the expression of renal protection factors. **(A)** Photomicrographs illustrating immunohistochemical staining of regeneration biomarker PCNA. Scale bar = 100 µm. **(B)** Quantification and graph of the number of PCNA positive cells from immunohistochemical staining. **(C)** Porcine kidney tissue lysates from four groups were subject to immunoblot analysis with specific antibodies against PCNA, Klotho and BMP-7. Expression levels of PCNA **(D)**, Klotho **(E)** and BMP-7 **(F)**, were quantified by densitometry analysis and then normalized with α-Tubulin. Data are means ± sem.*p < 0.05; **p < 0.01 versus sham controls.

Proliferating cell nuclear antigen (PCNA) is closely related to DNA synthesis in cells and its expression in renal tubular cells represents renal regeneration (Du et al., 2021). In this study, we examined the expression of PCNA in porcine kidneys by using both immunohistochemical staining and immunoblot analysis. As shown in Figures 6A,B, a small number of PCNA-positive tubular cells was observed in normal kidney tissue (sham group) by immunohistochemical staining, but this population of cells was significantly increased following I/R injury, and further elevated with exosome administration in pigs with AKI (Figures 6A–D). Similarly, PCNA was minimally detected in the sham-operated kidneys by immunoblotting and increased in the injured kidneys, and further up-regulated after injection of exosome. These results suggest that cell proliferation occurs in renal tubular cells following I/R injury, and administration of hUC-MSCs-derived exosomes further enhance this process.

Klotho and BMP-7 are two renoprotective factors and their deficiency is commonly observed in acute and chronic kidney injury (Jorge et al., 2019; Neyra et al., 2020; Sanchez-Niño et al., 2020). To understand the mechanisms of exosome-initiated renoprotection and regeneration, we examined their expression levels in the kidney. Our results showed that administration of MSCs-derived exosomes in part restored their expression in our I/R-induced AKI porcine model (Figures 6C,E,F), suggesting preservation and/or restoration of Klotho and BMP-7 expression may play an important role in promoting renal regeneration following injury.

### MSC-Derived Exosomes Inhibited the Expression of Pro-Inflammatory Factors and Infiltration of Macrophages in the Kidney of Miniature Pigs Following I/R Injury

**FIGURE 7:**
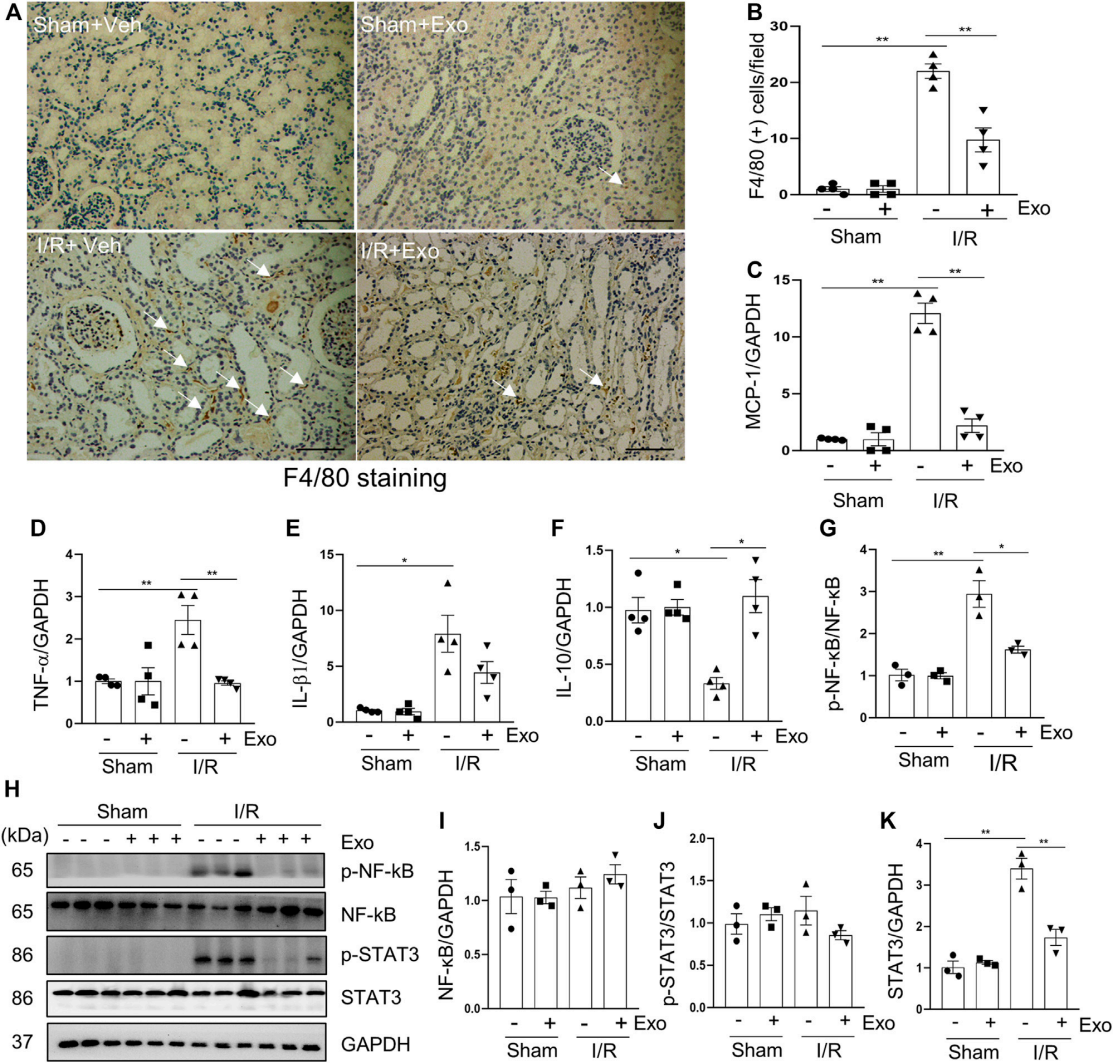
MSCs-derived exosomes inhibited production of pro-inflammatory factors. **(A)** Photomicrographs illustrating immunohistochemical staining of macrophage biomarker F4/80 Scale bar = 100 µm. Arrows point to F4/80-positive macrophages. **(B)** Quantification and graph of the number of F4/80 positive cells from immunohistochemical staining. RT-qPCR was applied to detect the expression level of the inflammatory factor MCP-1 **(C)**, TNF-α **(D)**, IL-1β **(E)** and IL10 **(F)**. **(H)** Porcine kidney tissue lysates from four groups were subject to immunoblot analysis with specific antibodies against phospho-NF-κb, NF-κB, phospho-STAT3 and STAT3. Expression levels of NF-κB **(I)** and STAT3 **(K)** were quantified by densitometry analysis and then normalized with GAPDH. The phosphorylation levels of NF-κB **(G)** and STAT3 **(J)** were calculated quantitatively by densitometry. Data are means ± sem.*p < 0.05; **p < 0.01 versus sham controls.

Inflammation is a major contributor to the development of AKI (Sølling et al., 2011). It has been reported that the infiltration of inflammatory cells to renal interstitium and the expression of pro-inflammatory factors increased, whereas the cytokines that suppressed inflammation were down-regulated in the kidney of rodent models of I/R (Semedo et al., 2009). F4/80 is a glycoprotein expressed only in macrophages, and is often used as a marker for mature macrophages (Haidl and Jefferies, 1996). Immunohistochemical staining demonstrated that the number of F4/80 positive cells was increased in the I/R injured kidney, and administration of MSC-derived exosomes reverses this response (Figures 7A,B). The results of real-time fluorescent quantitative PCR showed that renal mRNA levels of pro-inflammatory factors MCP-1, TNF-α, and IL-1β were increased in the I/R pigs, while the protective factor IL-10 was decreased slightly. Although the above-mentioned pro-inflammatory parameters were significantly reduced in the exosomes-treated group (Figures 7C–F), this treatment only restored renal IL-10 levels to a small degree, but did not reach a significant level.

The induction and resolution of inflammation are thought to be under control by several transcription factors such as NF-κB (nuclear factor-κB) and STAT3 (Signal Transducer and Activator of Transcription 3) (Fan et al., 2013). NF-κB and STAT3 pathways can be activated by their phosphorylation in response to diverse stimuli, including I/R injury (Linkermann et al., 2013). Consistent with what was observed in rodent models of I/R (Anders, 2018), phosphorylation levels of NF-κB and STAT3 were increased in the kidney of miniature pigs following I/R relative to shamed-operated pigs; exosomal treatment significantly inhibited their phosphorylation (Figures 7G–K).

Collectively, our results indicate that hUC-MSC-derived exosomes may alleviate renal injury by a mechanism involved in suppressing inflammatory responses during I/R.

### MSCs-Derived Exosomes Protect Against the Loss of Renal Angiogenesis in the Kidney of Miniature Pigs With I/R Injury

**FIGURE 8:**
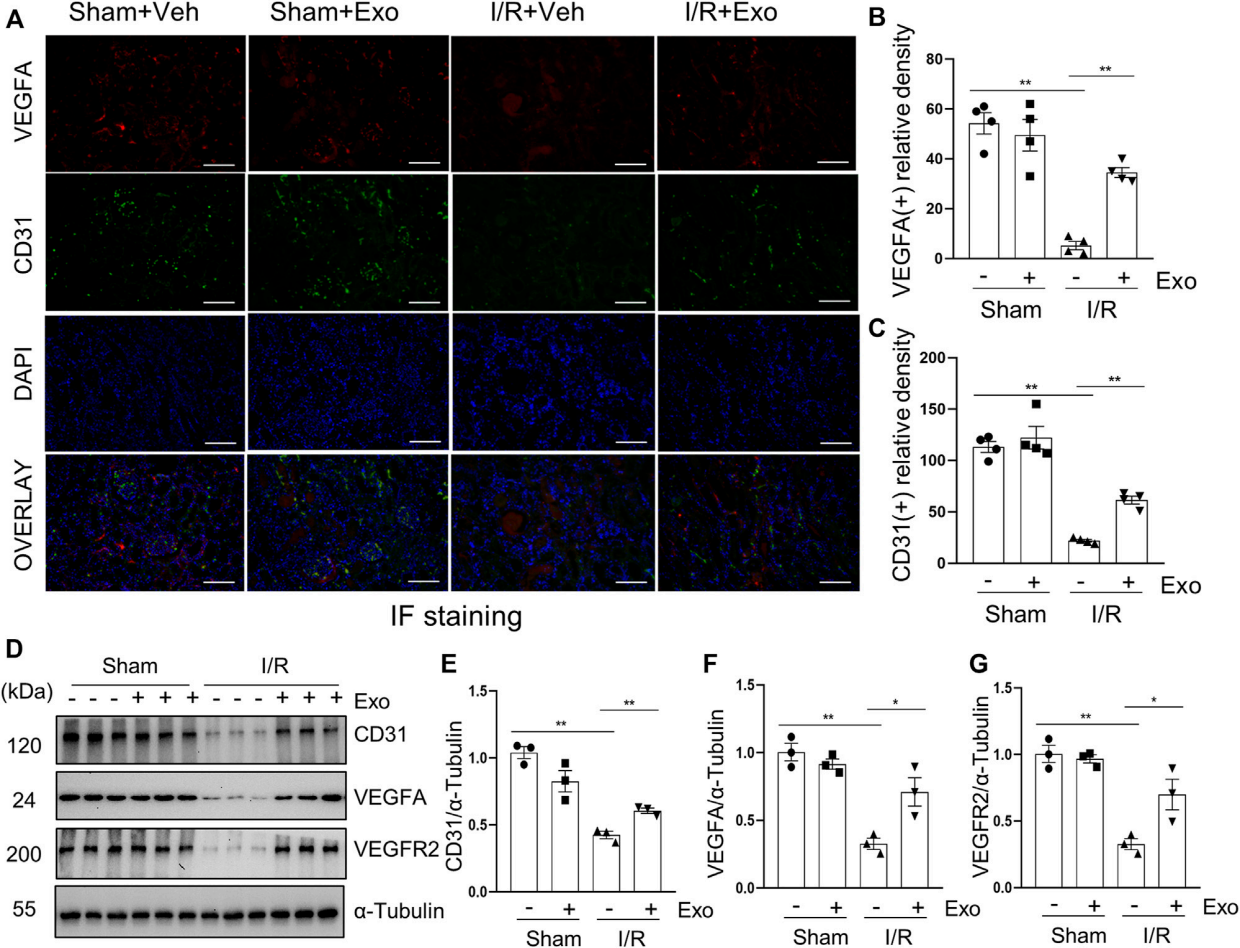
MSCs-derived exosomes restore loss of renal angiogenesis. **(A)** Photomicrographs illustrating co-immunofluorescence staining of endothelial markers CD31 and VEGFA. Scale bar = 200 µm. Quantification and plot of immunofluorescence density of VEGFA **(B)** and CD31 **(C)**. **(D)** Porcine kidney tissue lysates from each group were subject to immunoblot analysis with specific antibodies against CD31, VEGFA and VEGFR2. Expression levels of CD31 **(E)**, VEGFA **(F)** and VEGFR2 **(G)**, were quantified by densitometry analysis and then normalized with α-Tubulin. Data are means ± sem.*p < 0.05; **p < 0.01 versus sham controls.

It has been reported that diminished endothelial density is a common phenomenon in the kidney of I/R-induced AKI (Wu et al., 2021), whereas increased expression of VEGFA and in its receptor VEGFR2 is associated with renal neovascularization (Ricciardi and Gnudi, 2021). To further explore whether MSCs-derived exosomes would protect against AKI *via* regulating renal angiogenesis in the pig model of AKI, we examined the effect of exosomes on the expression of VEGFA and VEGFR2 in the kidney. Immunofluorescent staining showed that VEGFA and VEGFR2 as well as CD31, an endothelial biomarker, were all downregulated in the I/R injured kidney, and administration of MSCs-derived exosomes largely restored expression of all of them (Figures 8A–G). Therefore, MSCs-derived exosomes might also reduce renal injury through preventing the loss of endothelial integrity.

## Discussion

Currently, there are still no targeted treatments for AKI beyond supportive treatment and renal replacement therapy. One reason is that so far, small animal models (i.e., mouse and rat) were most used to explore the mechanism of renal injury and potentially develop novel therapeutic approaches for AKI. However, due to large differences between small animals and humans in many aspects such as size, immunity and genetics (Huang et al., 2021), data obtained from preclinical studies using small animals (i.e., mice and rats) are less successfully translated to clinical trials. As such, the US Food and Drug Administration (FDA) recently called for a therapeutic regime being tested in more than one animal model before submitting an investigative new drug application and at least one of the experimental models should be a large animal model. As such, in this study, we established an I/R induced AKI porcine model and investigated the efficacy of human umbilical MSCs-derived exosomes, and analyzed the possible mechanism(s) of action. We found that 120 min of renal ischemia followed by reperfusion can stably induce AKI in miniature pigs, and intravenous infusion of exosomes extracted from the supernatant of cultured hUC-MSCs exhibits a remarkable renoprotective effect on AKI in this model. Moreover, we demonstrated that the renoprotective effect of exosomes is associated with inhibition of renal tubular cell apoptosis/necroptosis and promotion of their proliferation, suppression of kidney inflammation and facilitation of angiogenesis. Thus, we have successfully established a stable large animal model of I/R-induced AKI and found that intravenous infusion of MSCs-derived exosomes may be a potential novel therapeutic strategy for AKI.

I/R-induced AKI is the most common cause of AKI. The previous literature offers different views on the appropriate duration of ischemia in pigs, with reports ranging from 15 to 120 min (Jayle et al., 2006; Giraud et al., 2011; Silberstein et al., 2013). Therefore, we set 3 protocols of different ischemia time to determine the optimal time. Our results show that both 60 and 90 min of ischemia followed by 72 h of reperfusion only caused mild kidney damage; renal function only slightly raised 24 h post occlusion of renal arterials and almost returned to the baseline level within 72 h 120 min is an ideal ischemia duration required for AKI modeling in pigs in view of renal dysfunction (as judged by SCr and BUN), pathological lesion, renal injury biomarkers and programmed cell death levels. The 2-h ischemia is much longer than what has been reported in other pig models in the literatures (Baldwin et al., 2004; Orvieto et al., 2005; Jayle et al., 2006; Brunswig-Spickenheier et al., 2010; Bechara et al., 2016; Damasceno-Ferreira et al., 2018). There might be two possible explanations. One is the ligation of isolated renal artery in this study could minimize the renal injury, instead of directly ligating the renal pedicle that include renal artery, vein and ureter as performed by other investigators (Baldwin et al., 2004; Orvieto et al., 2005; Silberstein et al., 2013). It has been reported that arteriovenous clamping (both renal artery and vein were clamped) caused more significant glomerular loss than clamping only the renal artery in a swine model (Bechara et al., 2016). Another explanation may be due to systematical injection of heparin sodium before ligation of the renal artery while none of other studies applied anticoagulation treatment (Jayle et al., 2006; Silberstein et al., 2013; Bechara et al., 2016). Application of heparin sodium may avoid formation of thrombus in the intrarenal blood vessels thereby ensuring perfect reperfusion in the whole kidney of individual pigs. Overall, we have successfully constructed a stable I/R-induced AKI porcine model and offered a piece of experience for other investigators to use such a large animal model.

In recent years, MSCs have been reported to protect against AKI as well as facilitate the repair process in rodent models (Kale et al., 2003; Morigi, 2004; Togel et al., 2005; Humphreys and Bonventre, 2008). Nevertheless, direct injection of stem cell therapy presented with potential risk of inducing delayed immune response and neoplasia (Cantaluppi et al., 2013). If the active ingredients in stem cells can be extracted as cell-free therapeutic agents, some of these risks can be avoided. The efficacy of MSCs-derived EV or exosomes in murine models of AKI have been reported by several laboratories (Alzahrani, 2019; Ullah et al., 2020a; Ullah et al., 2020b; Ullah et al., 2020c; Liu et al., 2020; Zhang C. et al., 2020). Many of these reports artificially modified exosomes and the contents inside (Zhang C. et al., 2020; Zhang Z. Y. et al., 2020), pretreated animals with pulsed focused ultrasound (Ullah et al., 2020c) or delivered exosomes with collagen matrix (Liu et al., 2020), to enhance or maximize efficacy. Since small animals are quite different from humans in terms of kidney anatomy, renal physiology, immune pattern and genetic background (Huang et al., 2021), this limited their translational significance. In this study, we used miniature pigs to investigate the therapeutic effect of MSCs-derived exosomes in AKI. Exosomes were detected in injured kidneys, but it is hard to decide whether they specifically homed to the damaged site or were just randomly distributed. Anyhow, MSCs-derived exosomes conferred potent renoprotective effects on porcine kidney, which is consistent with previous studies conducted in rodent models of AKI (Bruno and Camussi, 2014). After the infusion of exosomes, the renal function of miniature pigs was improved, renal pathological damage was reduced, the expression of kidney injury biomarkers was down-regulated, and levels of apoptosis and necroptosis were reduced. So far, reports on MSCs-derived exosomes therapy for AKI in large animals are rare. Only Eirin et al. (2017), reported that MSCs-derived EV attenuate renal inflammation 16-week post-administration in a metabolic syndrome and renal artery stenosis porcine model. Therefore, we are the first to report a potent therapeutic effect of human MSCs-derived exosomes on AKI in a large animal model.

The mechanism by which MSCs-derived exosomes protect against AKI were explored and associated with multiple mechanisms. We found that intravenous injection of MSCs-derived exosomes could reduce renal tubule injury as shown by decreased expression of NGAL and attenuate both apoptosis and necroptosis of renal tubular cells as demonstrated by reduced the number of Tunel-positive tubular cells, declined expression of cleaved caspase-3 and phosphorylated MLKL.

Proximal renal tubules show regenerative capacity, but in I/R-induced AKI, under the action of DNA damage, activation of inflammatory pathways and other mechanisms, renal tubular epithelial cells arrest in G1/S or G2/M phases of the cell cycle, resulting in decreased renal regenerative capacity, persistent deterioration of renal function, renal fibrosis, and progression to CKD (Moonen et al., 2018). PCNA is an important factor in DNA replication and repair, and is often used as an indicator of proliferation and tissue regeneration (González-Magaña and Blanco, 2020). Du et al. (2021) found that when exosomes overexpressing CD26 were isolated from the culture medium of a renal tubular epithelial cell line and treated intravenously in a mouse model of I/R-induced AKI, renal damage and histological evidence of AKI in the mice were alleviated; PCNA immunofluorescence staining showed that the cell proliferation originally induced by I/R injury was partially restored after exosome treatment. In this study, MSCs-derived exosomes were found to promote expression of PCNA in porcine kidneys by immunohistochemistry and immunoblotting analysis, suggesting the promotion of cell proliferation and tissue regeneration. In addition, we found that other regeneration-independent renal protective factors, such as Klotho, which has antioxidant and anti-aging properties, may also play a role in the exosomes-induced recovery from I/R-related kidney injury. In line with our observations, it was reported that transplantation of adipose-derived MSCs can ameliorate diabetic nephropathy in a mouse model, and the effect was reversed by siRNA-mediated knockdown of klotho (Ni et al., 2015). Therefore, we can reasonably speculate that MSCs-derived exosomes may have similar effects in AKI. Another renal protective molecule BMP-7 remains constitutively active in normal renal tubules; 120 min of ischemia followed by 72 h of reperfusion resulted in downregulation of it, while a dose of MSCs-derived exosomes could reverse this response. Thus, restoration of BMP-7 expression levels may be another mechanism by which MSCs-derived exosomes protect against AKI (Meng et al., 2013).

Moreover, administration of MSCs-derived exosomes resulted in suppression of elevated levels of pro-inflammatory cytokines MCP-1, TNF-α, IL-1β, increased macrophage infiltration, and up-regulated phosphorylation of NF-κB and STAT3 in the I/R injured kidneys. In contrast, the renal level of anti-inflammatory factor IL-10 was decreased following I/R injury and in part resumed after treatment with MSCs-derived exosomes. Finally, MSCs-derived exosomes were effective in promoting renal tubular cell proliferation in the injured kidney. Overall, the beneficial effects resulting from administration of exosomes may be associated with multiple aspects including immunomodulation, elevated ex-pression of renoprotective molecules and enhanced repair of injured renal tubular epithelial cells.

Endothelial dysfunction is a key mediator of oxidative stress and inflammation in AKI. It has been reported that transplantation of artificially modified apoptosis-resistant p53-silenced endothelial progenitor cells improves renal angiogenesis and vascularization in a mouse model (Kundu et al., 2021). In this study, I/R induced down-regulation of the expression levels of endothelial markers VEGFA, VEGFR2 and CD31 in the porcine kidney, and demonstrates that MSCs-derived exosomes can restore angiogenesis.

Nevertheless, there are some limitations in this study. First, the specific cargo inside the exosomes have not been identified in our study. Since exosomes contain a variety of active substances such as proteins, nucleic acids, lipids, etc., it is important to clarify the specific effective substances, which can enable us to develop a therapeutic strategy. In previous studies, some exosomal cargos such as miRNAs in particular, miR-125b-5p and microRNA let-7a-5p have been reported to promote renal repair following ischemic AKI in a murine model (Zhang C. et al., 2020; Cao et al., 2021). As such, both these two and other microRNAs need to be investigated in our model. Second, we only studied the short-term (72 h) effect of the exosomes on AKI in miniature pigs, the long-term effect remains unclear. Third, we have only investigated the effect of the exosomes on AKI induced by I/R, we do not know whether MSCs-derived exosomes have similar therapeutic effect in AKI induced by other insults such as sepsis and nephrotoxins.

In summary, we were the first to demonstrate that human MSCs-derived exosomes can alleviate I/R-induced AKI in a porcine model. The renoprotective effect of exosomes was associated with inhibition of renal apoptosis and necroptosis, attenuation of inflammation as well as promotion of renal repair and angiogenesis. Although multi-center randomized controlled clinical trials aimed at exploring the efficacy and safety of MSCs in patients with AKI after cardiac surgery are reported (Swaminathan et al., 2018), there is no clinical trial that directly uses exosomes as a cell-free treatment. The data obtained from this study provide reliable information for clinical trial design and guide new treatments for AKI using HC MSCs-derived exosomes.

## Data Availability

The raw data supporting the conclusion of this article will be made available by the authors, without undue reservation.
