# Supplementary material for: Mesenchymal Stem Cells-Derived Exosomes Ameliorate Ischemia/Reperfusion Induced Acute Kidney Injury in a Porcine Model
Source: Front Cell Dev Biol. 2022 May 24;10:899869. doi: 10.3389/fcell.2022.899869 (PMC9171021; doi:10.3389/fcell.2022.899869)
Supplement: Supplementary file 1 [file Presentation1.pdf]

SUPPLEMENTARY MATERIAL

Table S1: List of antibodies used in this study

Antibody	Cat. No.	Manufacturer
NGAL primary antibody	GTX60965	Genetex, San Antonio, TX, USA
GAPDH primary antibody	ARG10112	Arigo, Taipei, Taiwan, China
Cleaved Caspase-3 (Asp175) (5A1E) primary antibody	9664	Cell Signaling Technology, Danvers, MA, USA
Phospho-MLKL (Ser345) (D6E3G) primary antibody	37333	Cell Signaling Technology, Danvers, MA, USA
MLKL primary antibody	66675-1-Ig	Proteintech, Wuhan, China
Phospho-RIPK3 (phospho S232) primary antibody	ab195117	Abcam, Cambridge, UK
RIPK3 primary antibody	17563-1-AP	Proteintech, Wuhan, China
Antibody to CD9 (D3H4P)	13403	Cell Signaling Technology, Danvers, MA, USA
Antibody to CD81 (D3N2D)	56039	Cell Signaling Technology, Danvers, MA, USA
NF-κB p65 primary antibody	sc8008	Santa Cruz, CA, USA
Phospho-NF-κB p65 primary antibody	3033	Cell Signaling Technology, Danvers, MA, USA
STAT 3 (124H6) primary antibody	9139	Cell Signaling Technology, Danvers, MA, USA
Phospho-STAT 3 primary antibody	9145	Cell Signaling Technology, Danvers, MA, USA
F4/80 primary antibody	28463-1-AP	Proteintech, Wuhan, China
VEGFA primary antibody	MA1-16626	Invitrogen, Carlsbad, CA, USA
VEGFR2 primary antibody	26415-1-AP	Proteintech, Wuhan, China
CD31 primary antibody	PA5-32321	Invitrogen, Carlsbad, CA, USA
PCNA primary antibody	ARG62605	Arigo, Taipei, Taiwan, China
Klotho primary antibody	1854-1	Santa Cruz, CA, USA

BMP 7 primary antibody	4693	Cell Signaling Technology, Danvers, MA, USA
α-Tubulin primary antibody	2125	Cell Signaling Technology, Danvers, MA, USA

Table S2: Sequences of primers used in this study

Target	Gene ID	Upstream primer (5'-3')	Downstream primer (5'-3')
GAPDH	NM_001206359.1	TCTGGCAAAGTGGACATT	GGTGGAATCATACTGGAACA
TNF-α	NM_214022.1	GCCCCCAGAAGGAAGAGTTTC	CTGTCCCTCGGCTTTGACAT
IL-1β	NM_214055.1	GGCCATAGTACCTGAACCCG	GGTTTTGGGTGCAGCACTTC
IL-10	NM_214041.1	AGTGCCTTTAGCAAGCTCCAA	AGTCGTCATCCTGGAAGGTT
MCP 1	NM_214214.1	ATCAGCTCCCACACCGAAG	GGAGAATTAATTGCATCTGGCTGG

Table S3: Renal function parameters in pigs with different periods of ischemia

Group		Sham	I/R-60 min	I/R-90 min	I/R-120 min
SCr, μmol/L	Pre-operation	51.75±1.67	61.89±7.36	57.75±3.12	49.47±3.37
	24 h after operation	74.33±16.69	121.52±18.16	146.77±11.90	394.89±39.67
	48 h after operation	75.71±19.01	97.05±3.36	122.92±10.40	565.18±92.68
	72 h after operation	67.85±13.35	91.50±5.69	87.53±9.01	606.08±108.85
BUN, mmol/L	Pre-operation	3.99±0.43	5.18±0.63	5.76±0.47	4.13±0.64
	24 h after operation	6.65±1.69	10.84±1.24	14.77±1.67	23.34±3.98
	48 h after operation	5.05±1.07	6.77±0.70	8.92±0.33	28.05±11.37
	72 h after operation	3.29±0.81	4.92±0.63	5.12±0.36	30.30±12.94

Table S4: Renal function parameters in pigs with exosomes-treatment

Group	Body weight	SCr, μmol/L		BUN, mmol/L	
		D0	D3	D0	D3
Sham + vehicle	18.57±0.99	52.55±1.45	65.23±9.13	3.78±0.33	4.28±0.93
Sham + exosome	18.71±1.85	53.48±2.67	66.77±7.91	4.68±0.48	4.55±0.49
I/R + vehicle	18.31±1.30	48.12±3.26	601.08±67.85	4.25±0.53	30.45±12.86
I/R + exosome	18.73±0.39	61.24±6.24	204.99±32.06	5.19±1.1	11.37±3.10

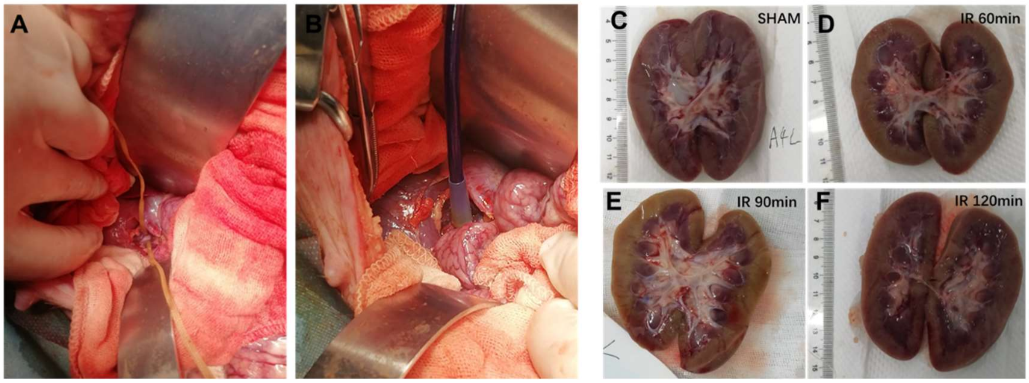

Figure S1: Photos of the surgical field during operation and general view of the kidney. (A) The left renal pedicle was exposed, the renal artery was separated, and the left renal artery was ligated with a non-destructive vascular ligation band; (B) The ligation band was passed through a Lumier trocar, and pressed down to block the left renal artery. Renal artery blood flow, and the trocar was fixed with vascular forceps, and the blood flow was blocked for 60, 90, and 120 minutes, and the blockage was released to restore tissue perfusion. view. (C-F) Coronal views of porcine kidneys in groups with different ischemia durations.
